# Somatic molecular analysis augments cytologic evaluation of pancreatic cyst fluids as a diagnostic tool

**DOI:** 10.18632/oncotarget.26999

**Published:** 2019-06-18

**Authors:** Ali Sakhdari, Parnian Ahmadi Moghaddam, Chi Young Ok, Otto Walter, Keith Tomaszewicz, Mandi-Lee Caporelli, Xiuling Meng, Jennifer LaFemina, Giles Whalen, Edward Belkin, Jaroslav Zivny, Wahid Wassef, Bruce A. Woda, Lloyd M. Hutchinson, Ediz F. Cosar

**Affiliations:** ^1^ University of Massachusetts Medical School, Department of Pathology, Worcester, MA, USA; ^2^ University of Massachusetts Medical School, Department of Surgery, Worcester, MA, USA; ^3^ University of Massachusetts Medical School, Department of Medicine, Worcester, MA, USA; ^4^ Massachusetts General Hospital, Department of Pathology, Boston, MA, USA; ^5^ MD Anderson Cancer Center, Department of Hematopathology, Houston, TX, USA; ^6^ University of Texas, Health Science Center, Department of Pathology, Houston, TX, USA

**Keywords:** pancreatic cyst classification, non-diagnostic cytology, molecular next generation sequencing

## Abstract

**Objective:** Better tools are needed for early diagnosis and classification of pancreatic cystic lesions (PCL) to trigger intervention before neoplastic precursor lesions progress to adenocarcinoma. We evaluated the capacity of molecular analysis to improve the accuracy of cytologic diagnosis for PCL with an emphasis on non-diagnostic/negative specimens.

**Design:** In a span of 7 years, at a tertiary care hospital, 318 PCL endoscopic ultrasound-guided fine needle aspirations (EUS-FNA) were evaluated by cytologic examination and molecular analysis. Mucinous PCL were identified based on a clinical algorithm and 46 surgical resections were used to verify this approach. The mutation allele frequency (MAF) of commonly altered genes (*BRAF*, *CDKN2A*, *CTNNB1*, *GNAS*, *RAS*, *PIK3CA*, *PTEN*, *SMAD4*, *TP53* and *VHL*) was evaluated for their ability to identify and grade mucinous PCL.

**Results:** Cytology showed a diagnostic sensitivity of 43.5% for mucinous PCL due in part to the impact of non-diagnostic (28.8%) and negative (50.5%) specimens. Incorporating an algorithmic approach or molecular analysis markedly increased the accuracy of cytologic evaluation. Detection of mucinous PCL by molecular analysis was 93.3% based on the detection of *KRAS* and/or *GNAS* gene mutations (*p* = 0.0001). Additional genes provided a marginal improvement in sensitivity but were associated with cyst type (e.g. *VHL*) and grade (e.g. *SMAD4*). In the surgical cohort, molecular analysis and the proposed algorithm showed comparable sensitivity (88.9% vs. 100%).

**Conclusions:** Incorporating somatic molecular analysis in the cytologic evaluation of EUS-FNA increases diagnostic accuracy for detection, classification and grading of PCL. This approach has the potential to improve patient management.

## INTRODUCTION

Recent advances in diagnostic imaging such as high-resolution ultrasonography (HR-US), computed tomography (CT), and magnetic resonance imaging (MRI) have increased detection rate of pancreatic cystic lesions (PCL) [1]. A substantial fraction (5–10%) of these cystic lesions are pancreatic cystic neoplasms (PCN), [2, 3] a significant majority of which are mucinous neoplastic cysts including intraductal papillary mucinous neoplasm (IPMN), and mucinous cystic neoplasm (MCN). IPMN and MCN carry malignant potential [4–9]. IPMN and MCN are mucinous precursor lesions for pancreatic ductal adenocarcinoma (PDAC), a highly aggressive tumor currently with a very low survival rate [10, 11]. Early detection of PDAC and its precursors has the potential to significantly increase survival rates [12, 13].

Accurate and early diagnosis of PCN is important for the appropriate management of patients and reduction of cancer-specific mortality. Separation of PCN from non-neoplastic PCL which are unlikely to require surveillance or surgery remains challenging. The current diagnostic approaches rely primarily on cytologic examination, tumor biomarker analysis of cyst fluid and high-resolution imaging [14]. The diagnostic power of imaging modalities including CT scan and HR-US is limited with an accuracy of 50–73% in differentiating neoplastic from non-neoplastic cysts [15] with considerable high inter- and intra-observer variability [16, 17]. The sensitivity of cytologic examination using EUS-FNA for the detection of mucinous precursor lesions is only 49 to 59% [18]. Carcinoembryonic antigen (CEA), among other biomarkers, has the best sensitivity (59–67%) for the detection of mucinous precursor lesions [18].

Guidelines have been published to assist in the diagnosis and management of PCL [19–21]. These incorporate clinical, laboratory and pathologic characteristics of PCL including patient age, clinical symptoms, pancreatic duct type and size, presence of a mural nodule, cytology of the cyst fluid, cyst size and diagnostic imaging characteristics [14, 19, 20, 22].

Molecular changes commonly found in PDAC, including abnormalities in *KRAS*, *GNAS*, *SMAD4*, *CDKN2A*, *BRAF*, *PIK3CA* and *TP53* genes can also be present in precursor lesions [23–25]. We hypothesized that incorporating the molecular analysis into PCL cytology specimens may increase the diagnostic accuracy and may help to identify patients who require surveillance or consideration for surgical management. This approach is especially important in paucicellular specimens which frequently yield a non-diagnostic cytology result yet may contain adequate material for molecular analysis [9, 26, 27].

In the current study, we show that molecular analysis of the cyst fluid has clinical utility in identifying gene mutations associated with neoplastic PCLs and improves the sensitivity/specificity of the clinical diagnosis particularly in non-diagnostic cytology specimens.

## RESULTS

### Molecular and cytology results for patients with surgical resections concur with algorithm-based classification

Of 278 patients with PCL and paired molecular studies, 46 (16.5%) underwent surgical resection at our institution (Supplementary Table 1). The surgical pathology diagnoses were as follows: IPMN (*n =* 19, 41.3%) (main duct [md] - and branch duct [bd]-IPMN), MCN (*n =* 9, 19.6%), PDAC (*n =* 8, 17.4%), SCA (*n =* 2, 4.3%), PNET (*n =* 3, 6.5%) and pseudocysts (*n =* 5, 10.9%) (Table 1). Among IPMN, MCN or PDAC diagnoses, cytology yielded a correct result in 15 of 36 (41.6%) whereas 21 cases were classified as either non-diagnostic (“ND”, *n =* 10) or negative (“NEG”, *n =* 11). Molecular analysis of preoperative cyst fluid from the IPMN/MCN/PDAC group with 36 surgical specimens in total, showed at least one mutation in *KRAS* or *GNAS* genes in 31 (86.1%), including 16 of the 21 (76.2%) cases incorrectly classified by cytology (“NEG”: *n =* 9, “ND”: *n =* 7). The sensitivity of molecular analysis in detecting mucinous PCN based on the presence of any mutation was 88.9% (32 of 36 cases) which is significantly higher than that of cytology (41.6%; 15 of 36 cases; *p =* 0.0001, Fisher’s exact test). This finding is similar to the algorithmic approach for classifying the mucinous PCL (Table 1 and Supplementary Data). Both SCA cases showed a *VHL* mutation. Mutations detected in cyst fluid were also detected in the surgical specimens (data not shown).

**Table 1:** Summarizes the pathologic, molecular and cytological characteristics of 46 surgical resection specimens of pancreatic cystic lesions (PCLs).

Cyst Type- grade	*KRAS*	*GNAS*	Other genes	Cytology result	Algorithm diagnosis	Molecular diagnosis^*^
**1- IPMN - LG**	p.G12V	NEG	NEG	**NEG**	**mPCN**	**mPCN**
**2- IPMN - LG**	p.G12V	p.R201C	NEG	**A/S**	**mPCN**	**mPCN**
**3- IPMN - LG**	p.G12V	p.R201C	NEG	**A/S**	**mPCN**	**mPCN**
**4- IPMN - LG**	p.G12V	p.R201H	NEG	**ND**	**mPCN**	**mPCN**
**5- IPMN - LG**	p.G12V	NEG	NEG	**ND**	**mPCN**	**mPCN**
**6- IPMN - LG**	p.G12R	NEG	NEG	**A/S**	**mPCN**	**mPCN**
**7- IPMN - LG**	p.G12R	p.R201H	NEG	**A/S**	**mPCN**	**mPCN**
**8- IPMN - LG**	p.G12A	p.R201H	NEG	**NEG**	**mPCN**	**mPCN**
**9- IPMN - LG**	p.G12R	NEG	NEG	**NEG**	**mPCN**	**mPCN**
**10- IPMN - LG**	NEG	NEG	NEG	**ND**	**mPCN**	**nmPCL**
**11- IPMN - LG**	p.G12V	p.R201C	NEG	**ND**	**mPCN**	**mPCN**
**12- IPMN - LG**	p.Q61H	p.R201H	NEG	**ND**	**mPCN**	**mPCN**
**13- IPMN - MG**	p.G12D	NEG	NEG	**NEG**	**mPCN**	**mPCN**
**14- IPMN - MG**	p.G12D	p.R201C	APC	**NEG**	**mPCN**	**mPCN**
**15- IPMN - MG**	p.G12V	p.R201C	***CDKN2A***	**POS**	**mPCN**	**mPCN**
**16- IPMN - MG**	p.G12A p.G12T	p.R201C p.R201H	NEG	**ND**	**mPCN**	**mPCN**
**17- IPMN - MG**	NEG	p.R201C	NEG	**ND**	**mPCN**	**mPCN**
**18- IPMN - HG**	p.G12V	NEG	***TP53, PIC3CA***	**A/S**	**mPCN**	**mPCN**
**19- IPMN - HG**	NEG	NEG	***BRAF***	**NEG**	**mPCN**	**mPCN**
**20- MCN - LG**	p.Q61H	NEG	NEG	**NEG**	**mPCN**	**mPCN**
**21- MCN - LG**	NEG	NEG	NEG	**ND**	**mPCN**	**nmPCL**
**22- MCN - LG**	p.G12D	NEG	NEG	**A/S**	**mPCN**	**mPCN**
**23- MCN - LG**	p.G12D	NEG	NEG	**NEG**	**mPCN**	**mPCN**
**24- MCN - LG**	NEG	NEG	NEG	**ND**	**mPCN**	**nmPCL**
**25- MCN - MG**	p.G12D	NEG	***TP53***	**A/S**	**mPCN**	**mPCN**
**26- MCN - MG**	p.G12V	NEG	NEG	**NEG**	**mPCN**	**mPCN**
**27- MCN - MG**	NEG	NEG	NEG	**NEG**	**mPCN**	**nmPCL**
**28- MCN - MG**	p.G12D	NEG	NEG	**ND**	**mPCN**	**mPCN**
**29- PDA**	p.G12R	NEG	***TP53***	**POS**	**mPCN**	**mPCN**
**30- PDA**	p.G12V	NEG	***TP53, SMAD4***	**POS**	**mPCN**	**mPCN**
**31- PDA**	p.G12D	NEG	***SMAD4***	**POS**	**mPCN**	**mPCN**
**32- PDA**	p.G12D	NEG	***RB1, SMAD4, PTEN***	**POS**	**mPCN**	**mPCN**
**33- PDA**	p.G12D	NEG	***TP53***	**POS**	**mPCN**	**mPCN**
**34- PDA**	NEG	p.R201H	***SMAD4, TP53***	**POS**	**mPCN**	**mPCN**
**35- PDA**	p.Q61C	p.R201C	***TP53, CDKN2A***	**POS**	**mPCN**	**mPCN**
**36- PDA**	p.G12D	p.R201H	NEG	**NEG**	**mPCN**	**mPCN**
**37- SCA**	NEG	NEG	***VHL***	**NEG**	**mPCN**	**nmPCL**
**38- SCA**	NEG	NEG	***VHL***	**NEG**	**nmPCL**	**nmPCL**
**39- PNET**	NEG	NEG	NEG	**A/S**	**mPCN**	**nmPCL**
**40- PNET**	NEG	NEG	***ATM***	**POS**	**mPCN**	**nmPCL**
**41- PNET**	NEG	NEG	NEG	**A/S**	**mPCN**	**nmPCL**
**42- Pseudocyst/CP**	NEG	NEG	NEG	**NEG**	**nmPCL**	**nmPCL**
**43- Pseudocyst/CP**	NEG	NEG	NEG	**NEG**	**nmPCL**	**nmPCL**
**44- Pseudocyst/CP**	p.G12D	NEG	NEG	**NEG**	**mPCN**	**mPCN**
**45- Pseudocyst/CP**	NEG	NEG	NEG	**NEG**	**nmPCL**	**nmPCL**
**46- Pseudocyst/CP**	NEG	NEG	NEG	**NEG**	**nmPCL**	**nmPCL**

(CP: chronic pancreatitis, IPMN: Intraductal papillary mucinous neoplasm, MCN: Mucinous cystic neoplasm, mPCN: mucinous pancreatic cystic neoplasm, nmPCN: non-mucinous pancreatic cystic neoplasm, PDA: Pancreatic ductal carcinoma, PNET: Pancreatic neuroendocrine tumor, SCA: Serous cystadenoma, A/S: atypical/suspicious, HG: high-grade, LG: low-grade, MG: intermediate grade, ND: non-diagnostic, NEG: Negative, POS: positive)

*Based on the presence or absence of any of the following genes: *BRAF, KRAS, GNAS, PIK3CA, CDKN2A, PTEN, SMAD4,* and *TP53.*

### Cytology diagnosis and case distribution

Among 318 PCL cases, cytology specimens were available for 309 cases. Cytology diagnoses were classified into four groups: non-diagnostic (“ND”, 28.8%, *n =* 89), negative for malignancy (“NEG”, 50.5%, *n =* 156), atypical/suspicious (“ATY/SUS”, 17.2%, *n =* 53,), and positive for malignancy (“POS”, 3.6%, *n =* 11). Patient demographics are given in Table 2.

**Table 2:** Shows clinical variables of 318 cases of pancreatic cystic lesions

Clinical variable	Pancreatic cystic lesions (*n* = 318)	Case Distribution: M = mucinous PCN NM = non-mucinous PCL
Cytology diagnoses:		
(n of 309, %)		
Non-diagnostic “ND”	(89/309, 28.8%)	**M**=45 (51%), **NM**=44 (49%) (*p =* 1.000)
Negative “NEG”	(156/309, 50.5%)	**M**=38 (24%), **NM**=118 (76%) (*p =* **0.0001**)
Atypical/suspicious “ATY/SUS”	(53/309, 17.2%)	**M**=53 (100%), **NM**=0 (0%) (*p =* **0.0001**)
Positive “ POS”	(11/309, 3.6%)	**M**=11 (100%), **NM**=0 (0%) (*p =* **0.0001**)
Female : male	162 : 156 (1.04)	**M**=0.87, **NM**=1.19 (*p =* 0.18)
Patient Age (year), Median (range)	61.5, (15 – 93)	**M**=66.8, **NM**=56.7 (*p =* **0.0001**)
CEA fluid concentration (ng/ml) Median (range)	1056.5, (0.1 – 11000)	**M**=2126.7, **NM**=188.9 (*p =* **0.0001**)
Cyst size (mm) Median (range)	33.6, (5 – 114)	**M**=30.2, **NM**=36.6 (*p =* **0.015**)
Amylase fluid concentration (u/L), Median (range)	6650.3, (2.4 – 24990)	**M**=5061.8, **NM**=7764.6 (*p =* **0.008**)
Cyst fluid viscosity (*n* = 275)	*Viscous* (***V***)=127 *Non-viscous* (***NV***)=148	***V***, **M**=91, **NM**=36 (*p =* **0.0001**) ***NV***, **M**=35, **NM**=113

The univariate analysis is performed between different characteristics in mucinous (M) and non-mucinous (NM) cases primarily classified by the proposed algorithm. Statistically significant differences are shown in bold.

### Cytology alone has a limited sensitivity for mucinous PCN

In our cohort, the algorithm identified a total of 147 mucinous PCN and 162 non-mucinous PCL (Table 2). The cytology results were compared with the algorithm-based classification. The cytology analysis alone for detecting a mucinous PCN has a sensitivity of 43.5%. Of the 147 mucinous PCN with available cytology diagnoses, cytology correctly identified 64 cases with a “POS” (*n =* 11) or “ATY/SUS” (*n =* 53) diagnosis but did not detect 83 cases (“ND”: *n =* 45, “NEG”: *n =* 38). Of 162 cases negative for mucinous PCN, cytology identified 118 cases as “NEG” but could not make a definitive diagnosis in 44 cases (all “ND”). While it was more likely for a cyst to be of non-mucinous subtype in the “NEG” group, the distribution of the mucinous PCN and non-mucinous PCL cases among non-diagnostic specimens were not significantly different (*p =* 1.00) (Table 2).

### Molecular analysis

#### Non-diagnostic cytology specimens contain *KRAS/GNAS* gene mutations

Of cases in the “ND” group 43.7% (38/87) showed mutations in *KRAS* and/or *GNAS* genes. This fraction is significantly higher than *KRAS* and/or *GNAS* mutation frequency observed in the “NEG” group (23.4%, 36/154, *p =* 0.002, Table 3). By contrast, the proportion of specimens with *KRAS* and/or *GNAS* mutations in “ATY/SUS” and “POS” cytology is significantly higher than the “NEG” or “ND” groups (85.5%, 53/62, *p =* 0.0001). At lower frequency, other gene mutations were also observed in “NEG” and “ND” groups [e.g *TP53* (1), *PIK3CA* (2), *ATM* (2), *APC* (2)]. Molecular analysis failed in a total of 4 cases in all groups.

**Table 3:** Shows *KRAS/GNAS* mutation prevalence across different cytology groups

Molecular analysis	*KRAS*, *n* (%)	*GNAS*, *n* (%)	*KRAS* or *GNAS*, *n* (%)
Cytology diagnoses			
Non-diagnostic “ND”	32/87 (36.8%)	15/51 (29.4%)	38/87 (43.7%)
Negative “NEG”	32/154 (20.8%)	14/81 (17.3%)	36/154 (23.4%)^*^
Atypical/suspicious “ATY/SUS”	43/51 (84.3%)	15/34 (44.1%)	44/51 (86.3%)
Positive “POS”	8/11 (72.7%)	3/9 (33.3%)	9/11 (81.8%)

*Note:* Patients with more than one mutation are represented in multiple categories.

(^*^the presence of a gene mutation is significantly lower in the negative “NEG” group compared to other groups, see text for further details).

### Mutant allele frequency (MAF) in non-diagnostic cytology group is comparable to atypical/suspicious cytology group

We compared the *KRAS* MAF (the most commonly mutated and tested gene in our cohort) between different cytology groups. The median *KRAS* MAF in “ND”, “NEG” and “ATY/SUS/POS” groups were 25.3% (range; 2%–54%), 17.8% (range; 1.5–52.5%) and 25.7% (range; 2%–88%) respectively. The *KRAS* MAF observed in the “ND” group approached the levels detected in the “ATY/SUS/POS” group. The difference between “NEG” and “ND” groups did not reach the statistical significance (*p =* 0.086).

**Figure 1:**
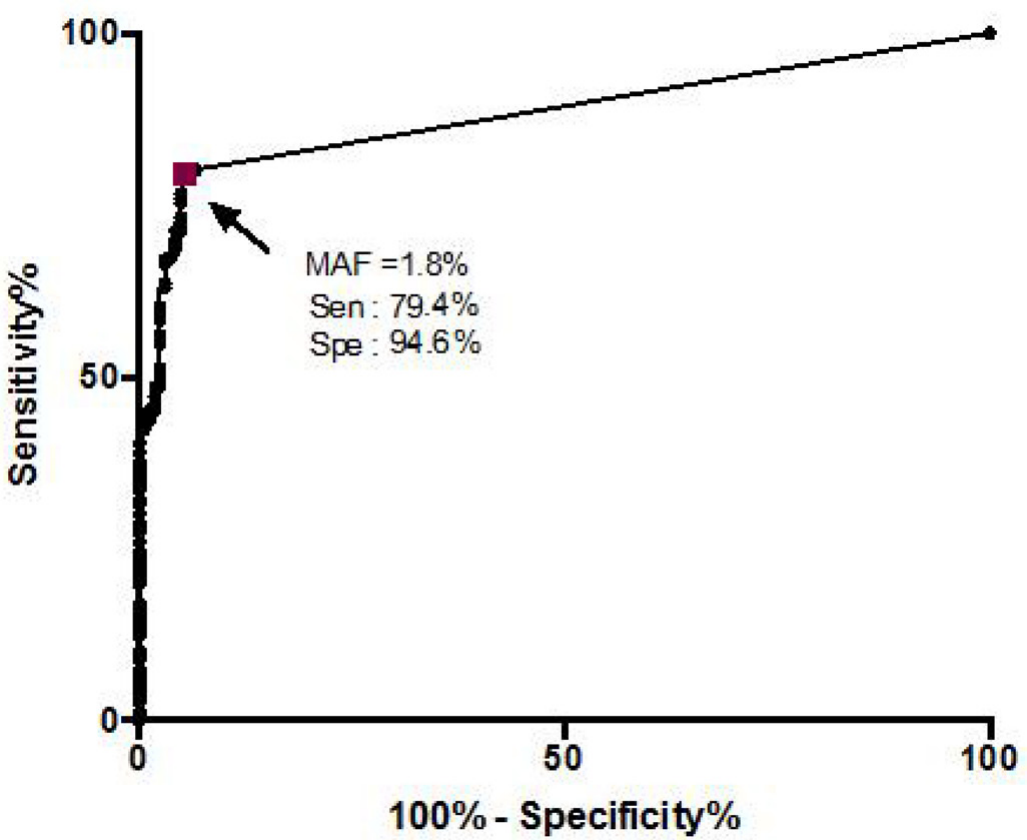
Receiver operating characteristic (ROC) curve plotted with *KRAS* mutation allele frequency (MAF) in pancreatic cystic lesions classified as either mucinous or non-mucinous based on the clinical algorithm. The purple square represents the MAF of 1.8% which showed the best combination of sensitivity and specificity for discriminating mucinous and non-mucinous cysts.

ROC curve was then utilized to calculate the *KRAS* MAF threshold for detection of mucinous PCN defined by the algorithm (Figure 1). Cytology specimens with mutations were divided into low and high MAF groups for *KRAS* based on the ROC-calculated threshold of 1.8%. This approach has the potential to improve specificity by excluding the incidental finding of precursor lesions such as PanIN. In specimens with a *KRAS* mutation, a low MAF was more commonly observed in the “NEG” compared to “ND” cytology (*p =* 0.02*,* Fisher’s exact test)*.* There was no difference in *KRAS* MAF between “ND” and “ATY/SUS/POS” (*p =* 1.000).

### Molecular analysis is more sensitive than cytology for detection of mucinous PCN

The molecular analysis results of all PCL cases were compared with algorithm-based classification. Of 318 PCL cases, molecular analysis for the *KRAS* gene was performed in 303 cases while multi-gene analysis was performed in a subset of 182 PCL cases. Any value of *KRAS* MAF had a significantly higher sensitivity for mucinous PCN than cytology analysis (82.4% vs. 43.5%, *p =* 0.0001) without adversely affecting the specificity (93.4%). Adding *GNAS* to the gene mutation analysis increased sensitivity to 93.3% (Table 4). Interestingly, 27 mucinous PCNs showed mutations in additional genes including 25 cases with additional *KRAS* and/or *GNAS* mutations (Table 5). Nevertheless, in this study expanding the molecular analysis with genes from the Ampliseq cancer hotspot panel v2 beyond *KRAS* and *GNAS* did not significantly improve the detection of mucinous PCN (data not shown).

**Table 4:** Illustrates the comparison between molecular analysis (*KRAS* or *KRAS/GNAS*) and cytology analysis for detection of a precursor mucinous pancreatic cystic lesion

Gene	*KRAS*	*KRAS* or *GNAS*	Cytology analysis
**Number of cases**	**(303)**	**(182)^a^**	**(309)**
**Sensitivity:**	82.4%^*^	93.3%^*^	43.5%
**Specificity:**	93.4%	83.9%	100%
**PPV:**	90.7%	84.7%	100%
**NPV:**	87.0%	92.9%	65.9%

^*^Sensitivity of molecular analysis is significantly higher than cytology (*p =* 0.0001).

^a^182 cases were tested by NGS for both *KRAS* and *GNAS* genes.

**Table 5:** Summarizes the non *KRAS/GNAS* mutations found in precursor lesions

Mutation type	Number of cases with mutation
*VHL*	8
*PIK3CA, TP53*	6
*ATM*	5
*SMAD4*	4
*CDKN2A*	3
*APC, FGFR3, JAK3*	2
*RB1, AKT1, ARID1A, MET, FBXW7, ERBB4, PTEN, FGFR2, KIT, SMO*	1

The cases with *VHL* gene mutation are likely to be serous cyst adenomas (SCA).

We next examined the impact of using the 1.8% cutoff to improve accuracy by separating “high” and “low” *KRAS* MAF. Of the non-mucinous PCL cases with a *KRAS* mutation, 9 of 11 (82%) had an MAF of more than 1.8% (media*n =* 5.3%; range = 1.47–18). Of the mucinous PCN cases with a *KRAS* mutation, 106 of 117 (93.2%) showed MAF of at least 1.8% (median *=* 26.8%; range = 1.2–87.7). Considering only the cases with a “high” *KRAS* MAF as mucinous PCN, a marginal change in the sensitivity (82.4% to 79.5%) and specificity (92.4% to 94.6%) was observed.

### Multiple mutations are common in pancreatic cystic lesions

Of PCL tested for *GNAS* and *KRAS* gene mutations, 19.8% (36/182) showed both *KRAS* and *GNAS* mutations. Thirty-five (97%) cases were predicted to have a mucinous PCN based on the algorithm-based classification. Among cases with double mutations, 33.3% (12/36) have at least 5% difference in MAF of two different genes. Of these, 83% (10/12) had a higher *KRAS* MAF (*p =* 0.003). In addition, 11% (14/127) of mucinous PCN cases carried more than one mutation in the same gene (*KRAS*: 10.4% [12/115], *GNAS*: 6.3% [3/47]). In 7 of 15 cases, the MAF differed by at least 5%. These findings may be due to presence of multiple cysts, or tumor heterogeneity or clonal evolution within a cyst (e.g. *KRAS* followed by *GNAS* mutation).

### The number and frequency of oncogenic mutations correlate with dysplasia

Of all the cases with available surgical resections, 36 cases were either pancreatic adenocarcinoma (PDAC, *n =* 8) or one of its precursors (IPMN, n = 19 and MCN, *n =* 9) (Table 1). Of 36 cases 17 (47%) were low grade (LG) lesions (IPMN, *n =* 12 and MCN, *n =* 5). These cases showed only *KRAS* (*n =* 14) and/or *GNAS* (*n =* 7) mutations. No double *KRAS* or double *GNAS* gene mutation was observed in these LG lesions. Of 9 cases (25%) with intermediate grade dysplasia (IPMN, *n =* 5 and MCN, *n =* 4), three (30%) showed mutations in three additional genes (*TP53, CDKN2A, APC*). This group also had one case with double mutation in both *KRAS* and *GNAS* (case #16, Table 1). All PDAC and mucinous PCN cases with high grade dysplasia (*n =* 10), showed additional gene mutations (*TP53, SMAD4, CDKN2A, BRAF, ARID1A, RB1, PTEN, PIK3CA*).

## DISCUSSION

Incorporation of multiple diagnostic modalities such as imaging techniques, cyst fluid chemical analysis and clinical findings, although helpful, do not provide early detection or accurate differentiation between non-neoplastic and neoplastic pancreatic lesions [15, 28]. Although rare, some mucinous PCNs carry a relatively high risk of malignancy. There remains a critical need for a better diagnostic tool to stratify patients for proper clinical management [29]. Recent studies indicate that molecular analysis of the *KRAS* and *GNAS* genes may be helpful in classifying PCL into neoplastic and non-neoplastic categories [30, 31]. Although many of these recent studies showed the added benefit of presence of molecular alterations in oncogenic driver genes, such as *KRAS*, the molecular analysis has not yet found its way into the standardized guidelines of pancreatic cystic lesions [32]. Here, we demonstrate the value of molecular analysis in patients with neoplastic PCL yet classified as “negative” or “non-diagnostic” by cytology. In a large cohort of PCL, we confirmed that cytologic evaluation has limited diagnostic value for low grade PCN [18]. In addition, we show that expanding the molecular panel beyond the *KRAS* and *GNAS* genes was helpful in identifying mucinous PCNs with moderate to high grade dysplasia.

Considering the surgical cases with mucinous PCN, there were a substantial number of cases (27.8%; 10 of 36) with non-diagnostic cytology. This limitation is similar to the previously published studies contributing to the low diagnostic sensitivity of cytology [33]. In contrast, molecular analysis showed a sensitivity of 88.9% (32 of 36). Furthermore, 90% of surgically proven PCN with non-diagnostic cytology showed at least one mutation in *KRAS* and/or *GNAS* genes (Table 1). The presence of these mutations and the MAF were significantly higher in the paucicellular non-diagnostic category than in the negative cytology group. These results suggest the molecular analysis is dependent on cell-free DNA, rather than cyst-fluid cellularity, and can serve as an important complement to morphologic evaluation particularly for pancreatic cystic lesions with non-diagnostic cytology.

In this study, we used an algorithmic approach based on international consensus guidelines to classify mucinous PCN and non-mucinous PCL [14, 19, 21, 34]. Surgical specimens (46/278, 16.5%) were used to assess the accuracy of the algorithm in classifying the PCL. Final pathologic diagnoses of surgical specimens were in agreement with the algorithm (89% concordance). When surgical specimens were used as the gold standard for the diagnosis of mucinous PCN, the sensitivity of cytology and molecular analysis was 41.7% and 88.9%, respectively. Cytology and molecular analysis of the cohort of 318 specimens classified using the algorithmic approach produced similar findings, yielding a sensitivity of 43.5% and 93.3%, respectively (table 4). To better assess the diagnostic value of cytology, paired analysis with surgical resection would be optimal; however, few patients need radical surgery. Nevertheless, our data support the use of the algorithm to classify PCL into mucinous and non-mucinous categories [35].

The algorithm disagreed with molecular analysis results in several cases. Eleven cases classified as non-mucinous PCL by the algorithm, showed *KRAS* (*n =* 10) or *GNAS* (*n =* 1) mutations. To improve accuracy of molecular analysis, we applied the cut-off of 1.8% *KRAS* MAF obtained from the ROC analysis. However, this cutoff value reclassified only 2 of 11 cases as non-mucinous PCL. While it is possible that these specimens represent true mucinous PCN misclassified by the algorithm, no follow-up surgical resection was available to support this possibility. In addition, 11 cases classified as mucinous PCN had *KRAS* MAF of less than 1.8%. These results suggest that use of a MAF cutoff for *KRAS* mutation may have a modest impact on specificity.

Our study demonstrates that gene mutation analysis can help discriminate between non-neoplastic and neoplastic cystic lesions. *KRAS* and *GNAS* gene analysis provided an overall sensitivity for detection of mucinous PCN, which was significantly higher than cytology alone (93.3% vs. 43.5%, Table 4). In the absence of *KRAS* or *GNAS* mutations, inclusion of genes such as *TP53, PTEN, SMAD4, PIK3CA, CDKN2A, BRAF* and *RB1* in the molecular analysis identified only one additional mucinous PCN. Although this finding suggests expanded gene panels may have limited utility for initial diagnosis, analysis of additional genes such as *RNF43, ATRX* and *DAXX,* which were not included in our study, may further improve diagnostic sensitivity [35, 36].

Regardless, an expanded gene panel may also be informative for dysplasia. Notably, among patients with surgical specimens, mucinous PCN with low-grade dysplasia showed mutations only in *KRAS* and/or *GNAS* genes. In contrast, mutations in additional genes were observed in mucinous PCN with moderate (3/9, 33%) and high grade (9/10, 90%) dysplasia. Both the number and the MAF of the of mutated genes increased with the grade of dysplasia (Table 1). This finding suggests acquisition of additional mutations is associated with progression from low grade to high grade dysplasia [37, 38]. The same mutations were detected in paired cytology and surgical specimens. Consequently, detection of such mutations in cytology samples, particularly in non-diagnostic specimens, may indicate the presence of a higher grade lesion and may warrant consideration for resampling or for surgical intervention in the appropriate context [35, 39]. For instance, MCN and IPMN with high grade dysplasia both require surgical excision; whereas low grade lesions may need more conservative approach, such as periodic observation for possible progression [40]. As none of the low grade mucinous PCN in our study had more than one *KRAS* and/or *GNAS* mutations, in the absence of other indications (e.g. grave clinical symptoms, positive cytology), periodic monitoring may be sufficient (Table 1). The absence of any mutations, nevertheless, did not preclude the need for surgery, since this molecular panel is not comprehensive and failed to detect PNET and 3 of 9 MCN.

Our study has both strengths and limitations. The incorporation of cytologic diagnosis into the algorithm [14] may artificially increase the specificity of cytology in this study. By contrast, molecular analysis was not included in the algorithm and consequently the sensitivity and specificity of molecular results are not affected. The choice of the cyst CEA level may also influence the accuracy of the algorithm. Based on previous studies, we used a CEA level of 192 ng/ml as a threshold for the classification of mucinous PCL [41, 42]. In our study ROC analysis for cyst CEA levels suggested that the cut off value of 124 ng/ml, rather than 192 ng/ml, would have the best combination of sensitivity and specificity for discriminating between mucinous and non-mucinous PCL (supplemental data). Nevertheless, the high concordance observed between the algorithm and surgical pathology diagnosis supports the use 192 ng/ml threshold.

This cohort was followed for a median of 3.3 years (range 1–8 years). Interestingly, 149 of the 318 specimens were classified as mucinous PCN by the algorithm, yet only 36 patients with mucinous PCN had surgical resection. In our study, it is possible that some patients may have been lost to follow up, since 12 patients had other mutations (i.e. *AKT1, APC, CDKN2A, FGFR2/3, PIK3CA SMAD4* or *TP53*) in addition to *KRAS* and/or *GNAS* mutations. Six of these patients had atypical/suspicious cytology diagnosis. Nevertheless, these results are consistent with the current guidelines [14, 21] and support the proposal that many mucinous PCN do not require surgical intervention at the time of diagnosis [35].

In summary, the high accuracy of somatic molecular analysis of pancreatic cyst fluid, particularly with non-diagnostic cytology specimens, to identify patients with neoplastic PCN which are more likely to benefit from surveillance, suggests that the standard of care should include somatic mutation analysis in the evaluation of pancreatic cystic lesions.

## MATERIALS AND METHODS

### Case selection

This study (IRB 14071) was approved by the institutional review boards of the University of Massachusetts Memorial Medical Center, Worcester, MA. From January 2008 to October 2015, 318 EUS-guided FNA of cyst fluid from 278 patients with pancreatic cyst(s) was evaluated during routine clinical testing, with follow up until December 2016. Nine specimens were not submitted for cytology analysis. Six specimens were not submitted for or failed molecular studies. Cyst fluid was subjected to chemical, cytopathologic and molecular genetic studies. Clinical, radiological and surgical follow-up data, if available, were recorded.

### Chemical analysis

The cyst fluid CEA (*n =* 224) levels were tested in most patients based on the strong clinical suspicion for PCN (Immunoassay (IA) - Quest Diagnostics™). The concurrent serum levels were also available for a select group of patients.

### Cytopathologic analysis

Cytology slides, prepared from the cyst fluid, were stained with the Papanicolaou (Pap) stain [43]. A Diff-Quik stain was also performed on air-dried smears. Based upon the cytology diagnosis, the cases were categorized into four groups: non-diagnostic (paucicellular or acellular), negative for malignancy, atypical/suspicious for malignancy and positive for malignancy [44]. To provide sufficient statistical power the atypical/suspicious/positive cases were grouped together for mutation allele frequency (MAF) analysis. These diagnostic categories are commonly used for pancreatic solid lesions. Although this multitiered reporting system can be applied to cystic lesions, it may be less reproducible due to heterogeneity and pauci-cellularity [44]. The diagnostic imaging, cyst fluid biomarker and endoscopic results and clinical impression were accessible prior to the cytopathologists’ diagnosis whereas the molecular studies were not.

### Molecular analysis

#### DNA extraction

To extract DNA from pancreatic cystic fluid, QIAamp Circulating Nucleic Acid Kit 55114 (QIAGEN Inc., Valencia, CA) was used. Extracted DNA was quantified with a UV NanoDrop 1000 spectrophotometer (DNA/RNA Calculator, Thermo Scientific, Waltham, MA) to determine the A260/A280 ratio. DNA quality was also evaluated using endpoint multiplex PCR to ensure DNA fragmentation was minimal. To extract DNA from surgical specimens, areas of tumor were identified by pathologists (AS, EFC, PAM) and manually micro-dissected from formalin-fixed, paraffin-embedded tissue. The collected cells were digested and genomic DNA was extracted as previously described [45].

#### Peptide Nucleic Acid (PNA) clamp PCR

Specific primer sets were used to amplify the region of the *KRAS* gene containing codons 12, 13 and 61, *GNAS* gene containing codon 201 and *BRAF* gene containing codons 598–602. PCR reactions were performed with and without a PNA clamp designed to block amplification of wild-type sequences (Supplementary Figure 1). In this real time PCR assay, the number of PCR cycles between the PCR reaction with and without PNA clamp was calculated to obtain a delta Ct (∆Ct). If the ∆Ct is ≥ 2 cycles above the 1% control, the result is reported as wild-type. If ∆Ct is < 2 cycles from the 1% control, the result is reported as mutant after BigDye Sanger sequencing confirmation (Thermo Fisher Scientific Inc., Waltham, MA). Comparing the value of ∆Ct in positive control/negative control (100%, 10%, 1% and 0% of mutation, respectively) with that of a patient specimen can be used to quantify the mutation levels i.e. if the ∆Ct equals the value of 100% positive control, the mutation frequency is 100%. PCR amplicons from the PNA reaction were digested with ExoSAP-IT® [46]. DNA products were then purified from the Sanger sequencing reaction and analyzed using capillary gel electrophoresis (ABI 3500xL Genetic Analyzer, Applied Biosystems, Foster City, CA) and fluorescence detection. This mutation analysis is able to detect the wild-type sequence and all known mutations associated with tested codons [47]. Results were interpreted using Soft Genetics Mutation Surveyor (SoftGenetics, State College, PA). The detection limit of the diluted positive control DNA in a background of wild-type alleles was 0.01%.

#### Next Generation Sequencing (NGS)

Next generation sequencing was performed as previously described using Ampliseq cancer hotspot panel V2 [48]. Amplicon libraries were created from genomic DNA (10 ng) according to the manufacturer’s protocol (Ion AmpliSeq Library Kit 2.0, Thermo Fisher Scientific Inc). The Ion CHEF Template System (Thermo Fisher Scientific Inc.) was used for emulsion PCR to amplify library DNA onto IonSphere Particles (ISPs) and load 318v2 chips. Sequencing was performed on an Ion Torrent PGM (IC200 Sequencing Kit, Thermo Fisher Scientific Inc. Waltham, MA) with coverage of 500–2500X. Variant calls from two software pipelines, Variant Caller V5 (Life Technologies, Inc) and NextGene V2 (SoftGenetics, Inc) were compared using a laboratory developed visual basic excel program. Variants with ≥10 reads and allele frequency greater than 1% were called positive although the limit of sensitivity is 0.2%. A manual review of nucleotide sequences was also performed for the genes frequently mutated in pancreatic cysts. After review, variants were confirmed as somatic mutations in the Catalogue of Somatic Mutations in Cancer (COSMIC) database [49] or ruled out as a known germline single-nucleotide polymorphisms (SNP) with the dbSNP database [50].

### Algorithm to identify mucinous neoplastic cysts

Cytologic diagnosis, cyst fluid CEA levels, cyst size, the presence of a mural nodule, fluid viscosity, and EUS clinical impression were used in a step-wise algorithmic approach to discriminate between mucinous and non-mucinous PCLs (supplemental data and Supplementary Figure 2 for further details).

### Receiver operating characteristics (ROC) curve analysis

ROC analysis was used to separate mucinous PCN from non-mucinous cysts based on 1) “high” and “low” *KRAS* MAF (defined as relative fraction of mutant PCR amplicon in the total DNA amplicon [51] and 2) cyst fluid CEA (see Supplemental Data) levels (ng/ml).

The *KRAS* MAF from cysts classified by the algorithm as non-mucinous (*n =* 165) and mucinous (*n =* 146) was plotted on an ROC curve to define a *KRAS* MAF threshold to most accurately separate non-mucinous from mucinous neoplastic lesion.

### Statistical analysis

Analysis of statistical difference between categorical data in our study was performed based on two-tailed Fisher’s exact test. Numerical data was analyzed using the Mann-Whitney Exact Test. Sensitivity, specificity, positive predictive value, and negative predictive value were calculated using standard 2 × 2 contingency tables. *p* value of less than 0.05 is considered significant. ROC calculation is performed using GraphPad PRISM 7 software.

## SUPPLEMENTARY MATERIALS



## Abbreviations

CEA: carcinoembryonic antigen
CT: computed tomography
COSMIC: Catalogue of Somatic Mutations in Cancer
EUS-FNA: endoscopic ultrasound-guided fine needle aspiration
HR-US: high resolution ultrasonography
MRI: magnetic resonance imaging
MAF: mutation allele frequency
NGS: Next Generation Sequencing
PCL: pancreatic cystic lesions
PCN: pancreatic cystic neoplasms
PNET: pancreatic neuroendocrine tumors
Pap: Papanicolaou
PNA: Peptide Nucleic Acid
ROC: Receiver Operating Characteristics
SNP: Single-nucleotide polymorphisms
IPMN: intraductal papillary mucinous neoplasms
MCN: mucinous cystic neoplasms
PanIN: pancreatic intraepithelial neoplasia
SPN: solid pseudopapillary neoplasms
SCT: serous cystic tumors
PDAC: pancreatic ductal adenocarcinoma
ND: non-diagnostic
NEG: negative for malignancy
A/S: atypical/suspicious
POS: positive for malignancy.
