# Supplementary material for: Somatic molecular analysis augments cytologic evaluation of pancreatic cyst fluids as a diagnostic tool
Source: Oncotarget. 2019 Jun 18;10(40):4026–37. doi: 10.18632/oncotarget.26999 (PMC6592293; doi:10.18632/oncotarget.26999)
Supplement: Supplementary file 1 [file oncotarget-10-4026-s001.pdf]

Somatic molecular analysis augments cytologic evaluation of pancreatic cyst fluids as a diagnostic tool

SUPPLEMENTARY MATERIALS

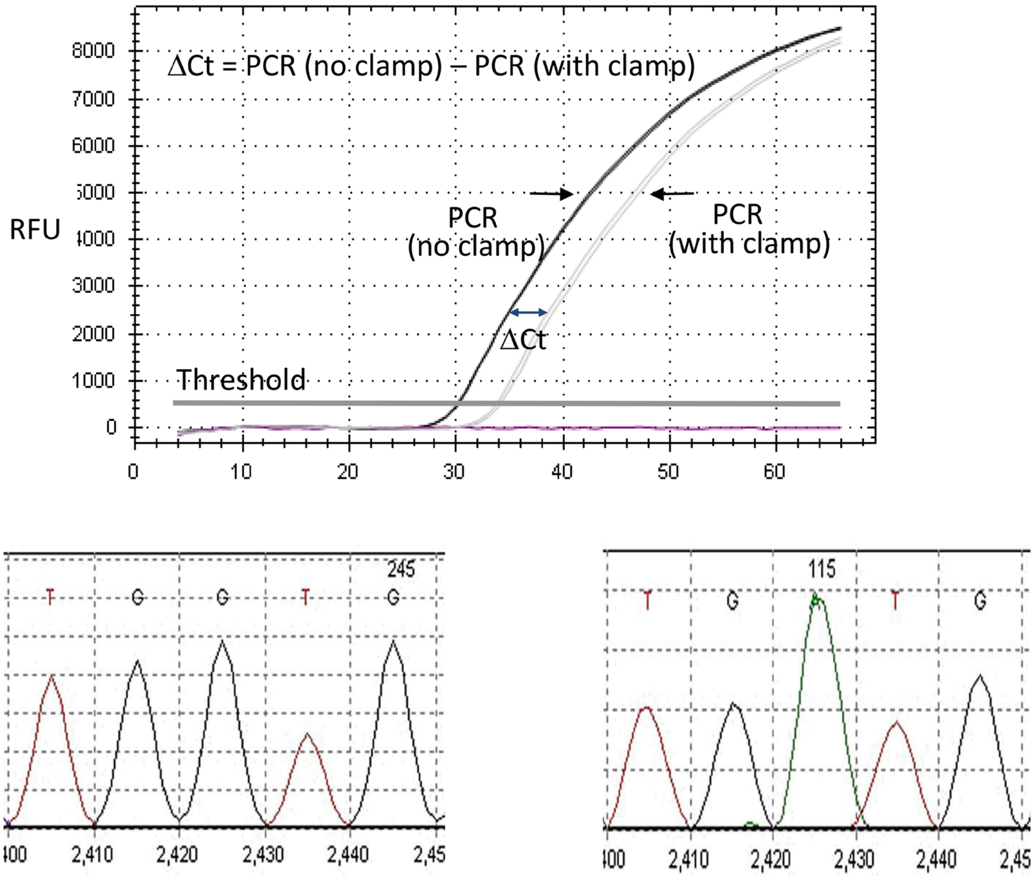

Supplementary Figure 1: *KRAS*-specific polymerase chain reaction (PCR) with or without peptide nucleic acid (PNA) clamp to enrich mutant *KRAS* codon 12/13 amplicons. ΔCt represents the difference between number of cycles needed to amply *KRAS* gene with or without the PNA clamp. If the ΔCT is ≥ 2 cycles above the 1% positive control, the result is reported as wild-type. If ΔCT is < 2 cycles from the 1% control, the result is reported as mutant after Sanger sequencing confirmation.

Algorithmic approach to identify mucinous PCN is highly sensitive

Most pancreatic cysts detected by diagnostic imaging are not neoplastic and should not be surgically resected. Consequently, the major parameters found in widely established guidelines were employed to identify the mucinous neoplastic cysts that represent the majority of cystic neoplasms. In order to verify the diagnostic accuracy of this approach, we compared the algorithm results with the surgical pathology diagnosis in a cohort of 46 cases. Forty-one cases were correctly classified as mucinous or non-mucinous cystic lesions achieving 89.1% concordance with the final surgical diagnosis. Five cases were incorrectly identified as mucinous neoplastic cysts by this algorithm. Three of these cases showed cystic pancreatic neuroendocrine tumors (PNET), misclassified due to a large size, clinical impression and abnormal cytology. One case of chronic pancreatitis was misclassified as mucinous due to a large size and highly suspicious clinical impression (weight loss and jaundice). Interestingly, this case showed very low levels of *KRAS* mutation in cyst fluid and surgical specimen, which may possibly be due to the presence of pancreatic intraepithelial neoplasia (PanIN) observed in the final resection specimen. The fifth case showed a serous cystadenoma (SCA) with *VHL* mutation in both cyst fluid and surgical resection specimen and was likely misclassified due to high fluid viscosity and large size (Supplementary Figure 2).

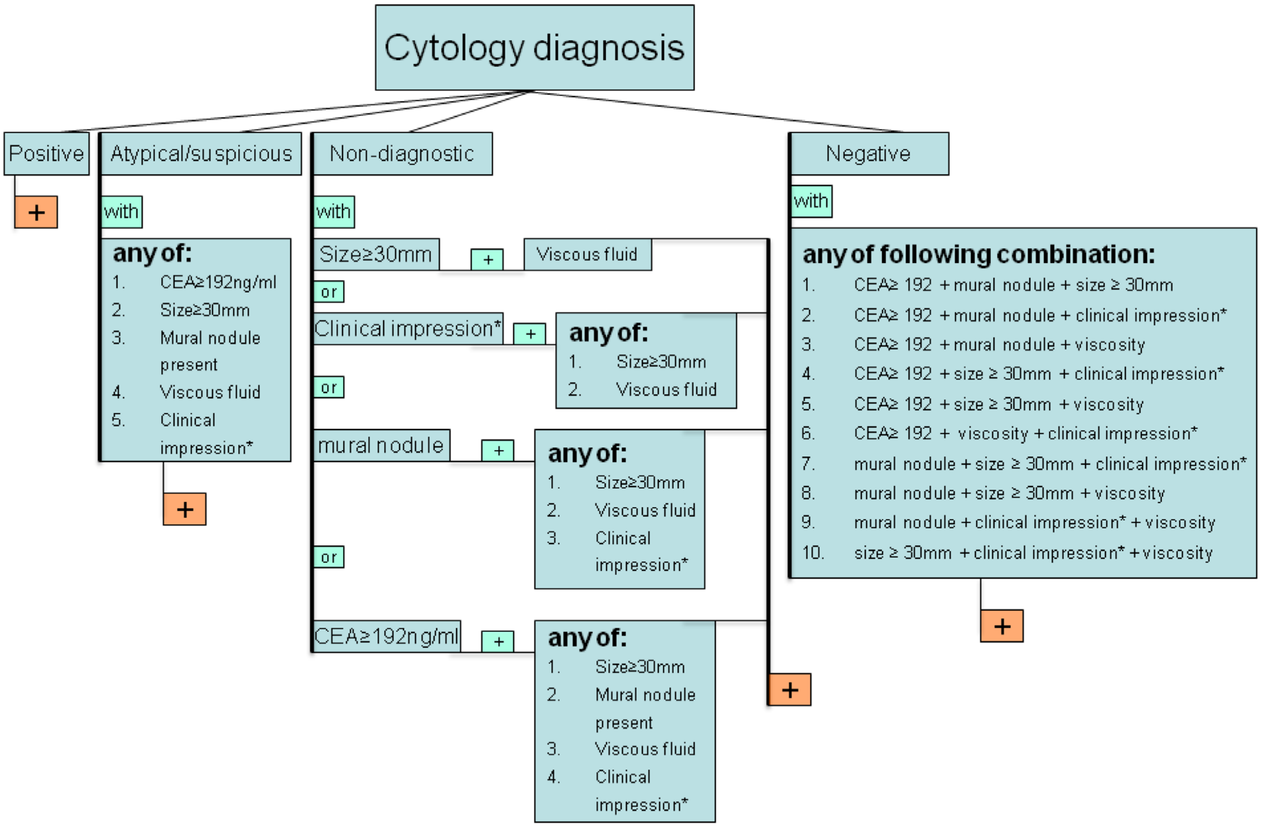

Supplementary Figure 2: Shown here is the algorithmic approach used for classification of pancreatic cystic lesions to mucinous (depicted as +) or non-mucinous. Cytology diagnoses were divided into four categories and based on the cytological diagnosis; multiple other clinical, laboratory or imaging characteristics were utilized to increase detection of a mucinous cyst. *Clinical impression represents the overall impression of clinical team and was mainly based on -presence or absence of jaundice, -ERCP finding, -cyst size between 10 mm to 29 mm, -location of the cyst, -presence or absence of recent acute pancreatitis, -other serum tumor markers (CA19-9, CA125) or -lipase and amylase. (CEA: carcinoembryonic antigen)

Cyst fluid CEA level is significantly higher in mucinous PCN

Cyst fluid CEA levels were available for 238 cases (131 non-mucinous PCL and 107 mucinous PCN). The numerical CEA levels (ng/ml) were significantly higher in the mucinous PCN ($p<0.0001$). We used a ROC curve to determine the CEA level that offers the best combination of sensitivity and specificity for detecting a mucinous PCN. The CEA level of 124 ng/mL provided an optimal sensitivity of 67.3% and specificity of 80.3% (Supplementary Figure 3). Notably, the CEA level of 192 ng/mL used in clinical practice and the diagnostic algorithm yielded sensitivity and specificity of 54.2% and 86.4% respectively and this was the value we used in the proposed algorithm.

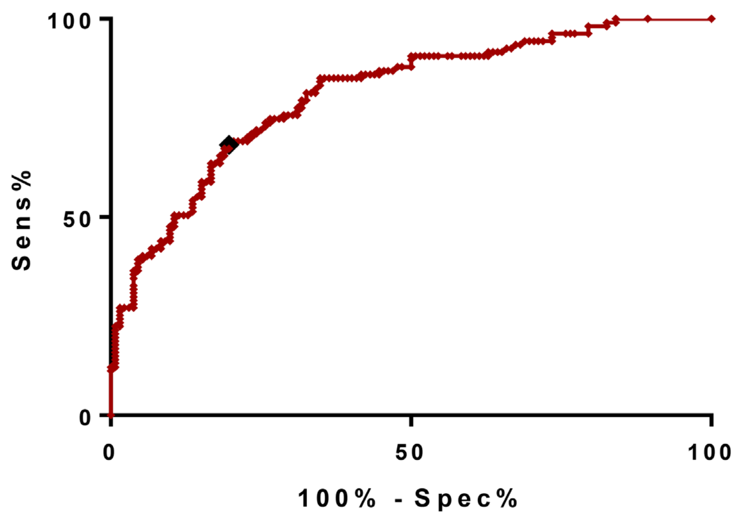

Supplementary Figure 3: Receiver operating characteristic (ROC) curve plotted with CEA levels of pancreatic cystic lesions classified as either mucinous or non-mucinous based on the algorithm. The black square represents the CEA value of 124 ng/ml which had the best combination of sensitivity and specificity for discriminating mucinous and non-mucinous cysts. Based on current clinical standards a value of 192 ng/ml was used in the algorithm (see Supplementary Figure 2).

Distribution of *KRAS* and *GNAS* gene codon mutations in cyst fluid

We observed 14 different types of *KRAS* mutations across different cyst fluids. The majority of these mutations were located in codon 12 (89%) followed by codon 61 (6%) and codon 13 (5%). The more commonly observed mutations are Gly12Val (p.G12V, 41%), Gly12Asp (p.G12D, 34%) and Gly12Arg (p.G12R, 15%) (Supplementary Figure 4). In the *GNAS* gene, the majority of mutations are in codon 201, specifically Arg201His (p.R201H, 52%) and Arg201Cys (p.R201C, 46%) (Supplementary Figure 5).

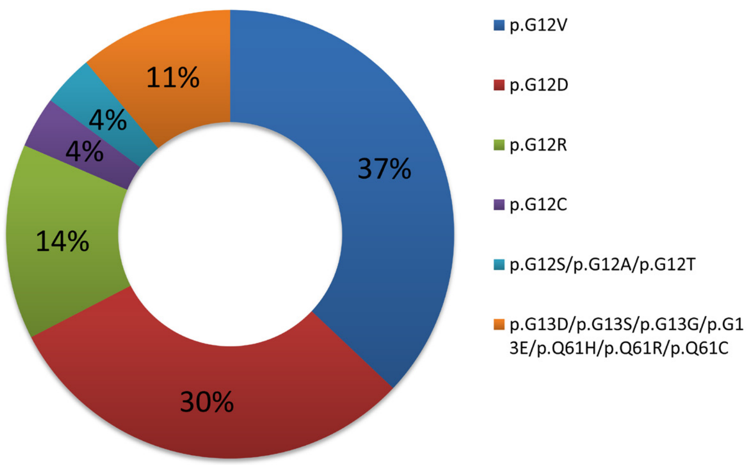

Supplementary Figure 4: Pie chart graph specifying different types of *KRAS* mutations observed in this study.

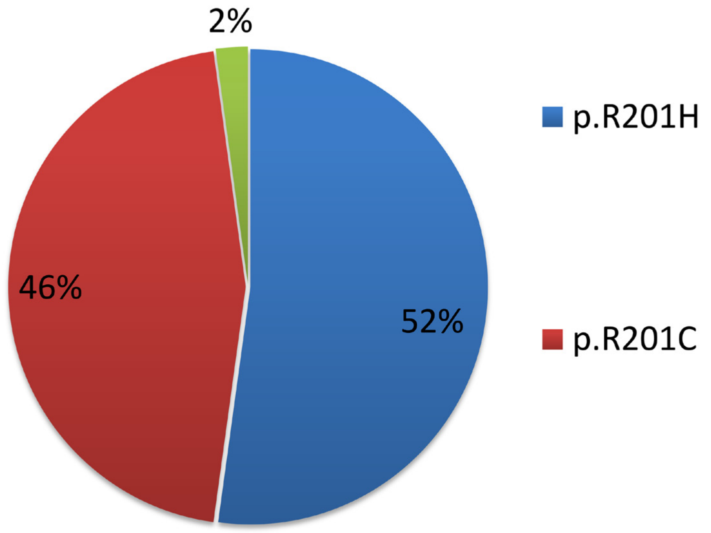

Supplementary Figure 5: Pie chart graph specifying different types of *GNAS* mutations observed in this study.

Supplementary Table 1: Patient cohort with final surgical resections

Diagnosis	IPMN (*n* = 19)	MCN (*n* = 9)	PDAC (*n* = 8)	SCA (*n* = 2)	PNET (*n* = 3)	CP (*n* = 5)	Total (*n* = 46)
Age, year<br>Median (range)	67<br>(34–77)	68<br>(23–84)	64<br>(43–87)	59<br>(50–69)	55<br>(35–67)	63<br>(45–71)	67<br>(23–87)
Male : female	14:5	4:5	4:4	0:2	1:2	2:3	25:21
CEA fluid concentration (ng/ml)<br>Median (range)	370<br>(2.7–11000)	501 (90–<br>11000)	NA	72<br>(2–142.6)	9.2<br>(0.7–17.7)	30<br>(1.2–192)	185.2<br>(0.7–11000)
Cyst size (mm)<br>Median (range)	34.5<br>(18–60)	43<br>(20–60)	40<br>(21–100)	52.5<br>(30–75)	20<br>(18–28)	35<br>(17–100)	35<br>(17–100)
Amylase fluid concentration (u/L),<br>Median (range)	185<br>(15–21000)	397 (24–<br>14400)	NA	71<br>(18–124)	968<br>(41–1895)	15360<br>(14400–21000)	397<br>(15–21000)
Cytology Dx							
Non-diagnostic “ND”	7	3	0	0	0	0	10
Negative “NEG”	6	4	1	2	0	5	17
Atypical/suspicious “ATY/SUS”	5	2	0	0	2	0	9
Positive “ POS”	1	0	7	0	1	0	9

Abbreviations: IPMN: Intraductal papillary mucinous neoplasm, MCN: Mucinous cystic neoplasm, mPCN: mucinous pancreatic cystic neoplasm, nmPCN: non-mucinous pancreatic cystic neoplasm, PDA: Pancreatic ductal carcinoma, PNET: Pancreatic neuroendocrine tumor, SCA: Serous cystadenoma.
